# Impact of blood factors on endothelial cell metabolism and function in two diverse heart failure models

**DOI:** 10.1371/journal.pone.0281550

**Published:** 2023-02-13

**Authors:** Young Song, Joseph Leem, Mehul Dhanani, M. Dan McKirnan, Yasuhiro Ichikawa, Julie Braza, Elizabeth O. Harrington, H. Kirk Hammond, David M. Roth, Hemal H. Patel

**Affiliations:** 1 Department of Anesthesiology and Pain Medicine, Anesthesia and Pain Research Institute, Yonsei University College of Medicine, Seoul, Republic of Korea; 2 Veterans Administration San Diego Healthcare System, San Diego, CA, United States of America; 3 Department of Medicine, UCSD School of Medicine, San Diego, CA, United States of America; 4 Department of Medicine, Brown University and the Vascular Research Laboratory, Providence VA Medical Center, Providence, RI, United States of America; 5 Department of Anesthesiology, UCSD School of Medicine, San Diego, CA, United States of America; Instituto Nacional de Cardiologia Ignacio Chavez, MEXICO

## Abstract

Role of blood-based factors in development and progression of heart failure (HF) is poorly characterized. Blood contains factors released during pathophysiological states that may impact cellular function and provide mechanistic insights to HF management. We tested effects of blood from two distinct HF models on cardiac metabolism and identified possible cellular targets of the effects. Blood plasma was obtained from daunorubicin- and myocardial infarction-induced HF rabbits (Dauno-HF and MI-HF) and their controls (Dauno-Control and MI-Control). Effects of plasma on bioenergetics of myocardial tissue from healthy mice and cellular cardiac components were assessed using high-resolution respirometry and Seahorse flux analyzer. Since endothelial cell respiration was profoundly affected by HF plasma, effects of plasma on endothelial cell barrier function and death were further evaluated. Western-blotting and electron microscopy were performed to evaluate mitochondrial proteins and morphology. Brief exposure to HF plasma decreased cardiac tissue respiration. Endothelial cell respiration was most impacted by exposure to HF plasma. Endothelial cell monolayer integrity was decreased by incubation with Dauno-HF plasma. Apoptosis and necrosis were increased in cells incubated with Dauno-HF plasma for 24 h. Down-regulation of voltage-dependent anion-selective channel (VDAC)-1, translocase of outer membrane 20 (Tom20), and mitochondrial fission factor (MFF) in cells exposed to Dauno-HF plasma and mitochondrial signal transducer and activator of transcription 3 (Stat3) and MFF in cells exposed to MI-HF plasma were observed. Mitochondrial structure was disrupted in cells exposed to HF plasma. These findings indicate that endothelial cells and mitochondrial structure and function may be primary target where HF pathology manifests and accelerates. High-throughput blood-based screening of HF may provide innovative ways to advance disease diagnosis and management.

## Introduction

Heart failure (HF) is a leading cause of morbidity and mortality worldwide; however, therapeutic intervention is limited. While some blood biomarkers have been used for diagnosis and monitoring therapeutic efficacies, there are limited targets for unbiased biomarkers that may impact specific pathophysiologic mechanisms [1]. Expansion of biomarkers to encompass and reflect more complex physiological changes may support precision diagnosis and comprehensive care. As impaired mitochondrial function is recognized as an important biological consequence of HF, many have proposed that mitochondrial dysfunction may be a meaningful diagnostic and prognostic indicator [2]. Evidence is growing to suggest that blood derived factors could be utilized to model systemic/organ mitochondrial (dys)function [3]. Blood plasma and serum can modulate mitochondrial function in donor systems [4–6]. Endothelial cells incubated with blood plasma from septic patients exhibit decreased mitochondrial respiration compared to the cells incubated with blood from control subjects [4, 5]. We also recently observed that blood serum from helium-conditioned mice could alter mitochondrial respiration in naive cardiac tissue and muscle cells [6]. Additionally, plasma from humans conditioned with helium reduces endothelial cell injury in response to hypoxia [7]. Such studies suggest that blood derived factors could be utilized to assess and screen complex physiology and pathophysiology using adaptable systems. A blood-based measure of mitochondrial function as a diagnostic tool has never been explored for HF.

The heart is comprised of a number of cell types and though the cardiac myocyte dysfunction ultimately drives HF pathophysiology, it is unclear if dysfunction in other cardiac cell types may be more primary and serve as a tool to screen HF pathophysiology in vitro. Therefore, we aimed to investigate the effects of HF-derived blood factors on cardiac mitochondrial respiration to define specific cardiac cell types that may be targeted. We first measured oxidative phosphorylation in naive mouse heart tissue after acute exposure to the blood plasma from rabbit models of daunorubicin- and myocardial infarction (MI)-induced HF. We next deconstructed the heart into its component cell types and probed the impact of HF-derived plasma on mitochondrial function in the individual cell types in vitro. Finally, we focused on endothelial cell biology as these were the most impacted cells.

## Materials and methods

### Blood plasma preparation

Given that rabbit models of human heart disease have similarities to human physiology and are practical alternatives to larger mammals, we used four groups of blood plasma obtained from previous studies in rabbit heart failure Models [8, 9]. Briefly, in the MI experiment, New Zealand White rabbits underwent thoracotomy wherein a cryo-probe cooled with liquid N_2_ was used to induce transmural MI by freezing anterior-lateral left ventricular (LV) free wall for 3 min. The rabbits that underwent thoracotomy and pericardiectomy alone were defined as MI-Control group (N = 6). Daunorubicin-induced cardiotoxicity consisted of 3 mg/kg of daunorubicin administered intravenously weekly to the New Zealand White rabbits for 9 weeks (Dauno-HF group, N = 7). Non-treated, age and sex-matched rabbits were defined as the Dauno-Control group (N = 8). Echocardiography at 12 week- and 9 week- follow-up periods in the MI and daunorubicin experiments showed decrease in ejection fraction of 41% and 46% in the HF models compared with the respective controls. Immediately after echocardiography, venous blood was obtained from the heart under anesthesia. The blood was collected and centrifuged at 2000 rpm for 15 min at 4’C. Plasma was transferred to a cryotube and then flash-frozen in liquid N_2_ and stored at -80°C until use. Both studies were approved by the Institutional Animal Care and Use Committee (IACUC) at the VA San Diego Healthcare System, and the rabbits were handled following the National Institutes of Health (NIH) Guide for the Care and Use of Laboratory Animals. The number of blood samples used for the present study were 8, 6, 7, and 8 in the MI-HF group, MI-Control group, Dauno-HF group, and Dauno-Contro groups, respectively.

A blood sample tube of each rabbit was labeled with letters (A, B, C, or D) instead of name of the groups and the number. All the experiments in the current study were performed by investigators who remained blinded to the blood groups until completion of data analysis.

### Respirometry of healthy mouse myocardium exposed to rabbit blood plasma

We explored the effects of HF blood plasma on healthy mouse myocardial mitochondrial function through high-resolution oxygraph-2K (O2K; Oroboros Instruments, Innsbruck, Austria). Experimental protocol was approved by the IACUC at the VA San Diego Healthcare System, and the mice were handled following the NIH Guide for the Care and Use of Laboratory Animals. We collected LV tissue from young (8–12 weeks of age) male C57BL/6J mice purchased from Jackson Laboratory (Bar Harbor, ME). Euthanasia was performed by cervical dislocation and heart tissue was excised and placed on ice-cold BIOPS solution composed of 2.77 mM CaK_2_EGTA, 7.23 mM K_2_EGTA, 5.77 mM Na_2_ATP, 6.56 mM MgCL_2_·6H2O, 20 mM taurine, 15 mM sodium phosphocreatine, 20 mM imidazole, 0.5 mM dithiothreitol, and 50 mM MES hydrate. Soaked in the BIOPS, endomyocardial tissue was obtained from the LV free wall and dissected manually along the longitudinal axis to get 0.6–1.2 mg fiber bundles using the needle-tipped forceps under magnification. Fibers were subsequently washed in ice-cold MIR06 solution, containing 0.5 mM EGTA, 3 mM MgCL_2_·6H2O, 60 mM lactobionic acid, 20 mM taurine, 10 mM KH_2_PO_4_, 20 mM HEPES, 110 mMD-sucrose, and 1 g/L fatty acid-free bovine serum albumin (BSA) for 10 min before the respirometry.

Quickly blotted and weighed, the fibers were put into the chamber filled with 2 mL of MIR06, and baseline respiration was measured after brief equilibration. Then, 10 μL rabbit blood plasma was added to the chamber, and after 10 min, substrate-uncoupler-inhibitor titration (SUIT) to measure mitochondrial oxidative phosphorylation (OXPHOS) was started. Each blood plasma sample was added to 2 chambers for the assay using the 2 different SUIT protocols simultaneously. Protocol 1 was as follows: 10 mM glutamate, 2mM malate, 2.5 mM adenosine diphosphate (ADP), 5 mM pyruvate (complex I respiration), 10 mM succinate (maximal OXPHOS), stepwise titration of 0.5 μM carbonylcyanide-4-(trifluoromethoxy)-phenylhydrazone (FCCP, maximal uncoupling), 3 μM rotenone for blocking complex (C) I (C II respiration), and 2.5 μM antimycin-A (non-mitochondrial respiration). Protocol 2 was all the same as protocol 1, except the addition of 0.2 mM octanoyl-l-carnitine, a medium-chain fatty acid, as a CI substrate to assess fatty acid oxidation (FAO). In both protocols, 10 μM cytochrome C was added just after pyruvate to test the integrity of the outer mitochondrial membrane, and samples with an increase in the respiration > 15% were excluded. All measurements were conducted at 37°C with O_2_ concentration above 200 μM. Data were expressed as oxygen flux rates (pmol·s^-1^ per mg of fiber wet weight). The blood samples were 6–8 in each group (biological replicate), and each blood sample was tested on the 3 different mice myocardium by the same protocol (technical replicate). Data were analyzed using the Oroboros Datlab 7.4 software (Oroboros Instruments, Innsbruck, Austria).

### Respirometry of cell lines exposed to rabbit blood plasma

Bioenergetic profiling of cells to test the impact of blood plasma was performed by mito-stress test on a Seahorse extracellular flux analyzer (Agilent, North Billerica, MA). We used 4 different immortalized cell lines to explore which components of myocardium could be responsible for the altered heart tissue respiration as follows: H9C2 myoblast (ATCC, CRL-1446), Ea.hy926 endothelial cells (ATCC, CRL-2922), NIH/3T3 fibroblast (ATCC, CRL-1658), and RAW 264.7 macrophage (ATCC, TIB-71), all of which were grown in DMEM (Gibco, Life Technologies) supplemented with 10% fetal bovine serum (Gibco, Life Technologies), 2 mmol/L glutamine, and penicillin-streptomycin (100 IU/mL, Sigma-Aldrich). Analysis of blood plasma effects on cellular metabolism was performed as previously described [6]. Briefly, after achieving optimal cell seeding density and concentration of the assay agents from the preliminary experiments on each cell type, cells were seeded on a 96-well seahorse assay plate and grown for 20 h. The medium was then replaced with the Seahorse XF base medium, and cells were incubated in a 37°C incubator without carbon dioxide (CO_2_) for an hour. After measuring baseline oxygen consumption rate (OCR), rabbit blood plasma (1% final concentration) was added to the cells and incubated for 30 min with OCR measure. Then, oligomycin (a complex V inhibitor), FCCP (uncoupler), and rotenone mixed with antimycin A (complex I and III inhibitors) were sequentially added to measure adenosine triphosphate (ATP)-linked respiration, maximal respiration, and non-mitochondrial respiration, respectively. Each of the two-matched groups from the same rabbit study (HF and the corresponding control) was simultaneously assessed on the same plate. The blood samples were 6–8 in each group (biological replicate), and each plasma sample was injected into 6–7 wells per plate as a technical replicate. The OCR values were normalized to the baseline values and expressed as %.

### Ultrastructure of endothelial cell mitochondria exposed to rabbit blood plasma

Transmission electron microscopy was used to examine morphological changes in endothelial cell mitochondria. Ea.hy926 cells were seeded onto 6-well plates and grown to ~75% confluence. After serum starvation for 2 h, experimental blood plasma was added to the medium (1% final concentration). After 30 min, cells were fixed with 2.5% glutaraldehyde for 24 h at 4°C and then moved to 0.1 M cacodylate buffer at room temperature. Following post-fixing with 1% OsO_4_ in 0.1 M cacodylate buffer (1 h), cells were dehydrated in an increasing ethanol series and embedded in LX-112 (Ladd Research, Williston, VT). Ultrathin sections were collected on copper grids, counterstained with uranyl acetate and lead citrate, and inspected using the Jeol 1200 EX-II electron microscope (Jeol Ltd., Akishima, Japan) with the 8000x and 25000x of magnification. The Eagle 4k HS digital camera (FEI, Hillsboro, OR) was used to acquire and capture images.

### Permeability of endothelial cell monolayer exposed to rabbit blood plasma

Changes in endothelial monolayer permeability by blood plasma was assessed using the electrical cell impedance sensor (ECIS) technique (Applied Biophysics, Troy, NY). Five samples were randomly selected from each blood plasma group and used for this experiment. Rat heart microvascular endothelial cells (Vec Technologies, Rensselaer, NY) were seeded on collagen-coated 8 well ECIS arrays (8W10E+, PC; 8W1E, PET) at a density of 35x10^4^ cells/well. After a baseline read period, cell monolayers were treated with vehicle (phosphate buffer solution, PBS) or a 1% final concentration of rabbit blood plasma, and resistance was measured over 24 h. Data were normalized to the moment diluent was added (time 0). Resistance at 5 h post-treatment was subtracted from time 0 and expressed as ’drop-in resistance at 5 h post-treatment’. Each ECIS sample was run in triplicates, except the treatment of the vehicle in duplicate.

### Viability of endothelial cells exposed to rabbit blood plasma

Effects of the HF blood plasma (1 h and 24 h exposure) on the endothelial cell viability was assessed by annexin/PI apoptotic assay. Five samples were randomly selected from each blood plasma group and used in this assay. Ea.hy926 cells were seeded onto 6-well plates and grown for 24 h at 37°C. Then the cells were serum-starved for 2 h, followed by blood plasma addition to the medium (1% final concentration). After 1 h incubation with the blood plasma, cells were harvested and washed three times with ice-cold 1x PBS and resuspended in 1x annexin binding buffer (Biovision, Mountain View, CA). 1x10^5^ of cells were collected and incubated with 5 μL of Annexin V-Cy5 (Biovision) and 1 μL (100 μg/mL) of propidium iodide (PI, Invitrogen^TM^) in the dark for 15 min at room temperature, after which 1x annexin binding buffer was added to make a total volume to be 300 μL. The percentages of apoptotic and necrotic cells were measured by flow cytometry (BD FACS CantoTM, Becton Dickinson, Franklin Lakes, NJ). The same protocol was applied to the cells, which were incubated with experimental blood plasma for 24 h. The experiments were performed in triplicates.

### Endothelial cytotoxicity of rabbit blood plasma

Cytotoxic effect of HF blood plasma on Ea.hy926 cells (1 h and 24 h exposure) was evaluated by determining lactate dehydrogenase (LDH) release from cells using the CyQUANT™ LDH Cytotoxicity Assay (Invitrogen; Thermo Fisher Scientific, Inc., Waltham, MA) according to the manufacturer’s instructions. Briefly, Ea.hy926 cells were seeded at a density of 2x10^4^ cells/well in triplicates on a 96-well plate. After 36 h, medium was changed to serum-free media for 2 h, followed by addition of 10 μL of rabbit blood plasma, pure water, or empty to observe plasma-treated LDH activity, spontaneous LDH activity, and maximum LDH activity, respectively. After 1 h, 50 μL per well of supernatant was transferred into a new 96-well plate, and 50 μL of the reaction mixture was added to each well. Following 30 min incubation in the dark, the stop solution was added, and the absorbances at wavelength 490 nm and 680 nm were measured using the Spark^®^ multimode reader (Tecan, Austria). The LDH activity was determined as a subtraction of the 680 nm absorbance from the 490 nm absorbance. A % cytotoxicity was defined as (plasma-treated LDH activity—spontaneous LDH activity) / (maximum LDH activity—spontaneous LDH activity) x 100 (%). The assay for the cells exposed to the rabbit blood plasma for 24 h was performed in the same way as above, except that duration of the first cell culture was 24 h, instead of 36 h. The experiments were performed in triplicates.

### Western blot

Effects of blood plasma on the expression of components in endothelial mitochondria was assessed by western blot analysis. Five samples were randomly selected from each blood plasma group and used in this assay. Ea.hy926 cells were seeded to 12-well plates and grown ~75% confluence. After serum starvation for 2 h, experimental blood plasma was added to the medium (1% final concentration). After 1 h incubation, cell lysate extraction was performed in Radio-Immunoprecipitation Assay (RIPA) buffer (50 mM Tris-HCl, pH 8.0, 1% NP40, 150 mM NaCl, 0.5% deoxycholate, 0.1% SDS) supplemented with 10 μl/ml protease and phosphatase inhibitor cocktail (#78443, Thermo Scientific). Equal amounts of proteins, determined by Bicinchoninic Acid (BCA) protein assay (Pierce, Rockford, IL), were separated by sodium dodecyl sulfate—polyacrylamide gel electrophoresis (SDS-PAGE) and transferred to polyvinylidene difluoride (PVDF) membranes (Millipore Co., Bedford, MA, USA) by electroelution. The membranes were blocked with 5% non-fat milk (Bio-Rad Laboratories, Hercules, CA) and incubated with primary antibodies against optic atrophy (OPA)-1, dynamin-related protein (DRP)-1, voltage-dependent anion-selective channel (VDAC)-1, translocase of outer membrane (Tom)20, signal transducer and activator of transcription (Stat)3, and mitochondrial fission factor (MFF) in 5% bovine serum albumin overnight at 4°C. Monoclonal anti-glyceraldehyde 3-phosphate dehydrogenase (GAPDH) antibody and rabbit polyclonal anti-cytochrome c oxidase (COX) IV antibody were used to control whole cell protein and mitochondrial loading, respectively. After washing and 1h incubation with horseradish peroxidase (HRP)-conjugated secondary antibody in blocking solution, the chemiluminescent signal was detected by enhanced chemiluminescence (ECL) reagent. Signals were visualized with ECL reagent (Amersham Pharmacia Biotech, Piscataway, NJ, USA) and analyzed by densitometry (Syngene, Cambridge, United Kingdom).

### Statistical analysis

All data are presented as mean and standard error of the mean (SEM). The normality of data was determined via the Shapiro-Wilk test. Unpaired Student’s t-test and 2-way analysis of variance (ANOVA) with Bonferroni post-hoc analysis were used to compare the effects of HF plasma and the corresponding control plasma where appropriate. GraphPad Prism 8 software (GraphPad Software, Inc., San Diego, CA, USA) was used for all statistical analysis and statistical significance was defined as p < 0.05 or α = 0.05.

## Results

### Effects of HF blood plasma on the respirometry of healthy myocardium

**Fig 1:**
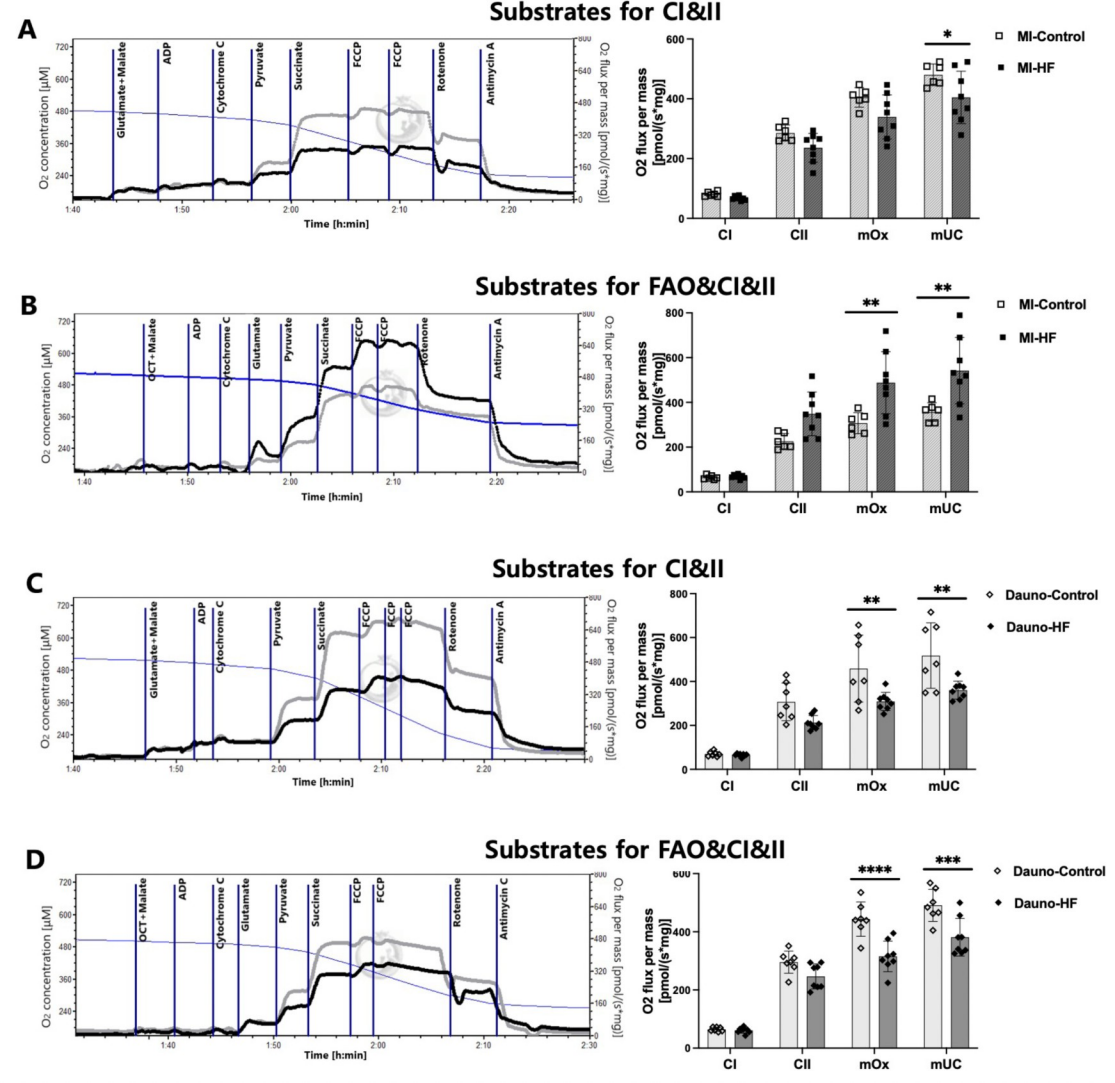
High-resolution respirometry analysis of naive mouse left ventricular myocardial fibers exposed to heart failure rabbit blood plasma. Naive mouse left ventricular tissue was incubated with the blood plasma from the heart failure (myocardial infarction- and daunorubicin-induced) rabbits or corresponding control rabbits in the O2K chamber for 10 min and assessed for respiration. The left panels show representative respirometry traces of oxygen flux (grey plots-Control, black plots-HF) in response to sequential administration of substrates and inhibitors. Respiration was measured as oxygen flux (= oxygen consumption rate) per tissue mass calculated by the negative slope of oxygen concentration with time (blue plots). The right panels are quantified respiratory function of complex I (CI) and II, maximum oxidative phosphorylation capacity (mOx), and maximum uncoupled capacity (mUC). Two different protocols of substrates were utilized—(A) and (C); glutamate and malate for CI, (B) and (D); octanoyl-l-carnitine (OCT), malate, and glutamate for CI and fatty acid oxidation (FAO). Assays utilized n = 6–8 plasma samples per group and 3 technical replicates per each plasma sample. Data are presented as means and SEM. Two-way ANOVA with Bonferroni post-hoc test was used. *P < 0.05, **P < 0.01, ***P < 0.001, and ****P < 0.0001 of HF vs. Control. Dauno = Daunorubicin; FCCP = carbonyl cyanide *p*-trifluoro methoxyphenylhydrazone; HF = heart failure; MI = myocardial infarction.

Mouse LV myocardial tissue exposed to the MI-HF blood plasma showed 16% lower maximal uncoupling (P = 0.032) than tissue exposed to the MI-Control blood plasma following the glutamate, malate, ADP, and pyruvate as substrates for CI (Fig 1A), 59% higher maximal OXPHOS (P = 0.0018), and 52% higher maximal uncoupling (P = 0.0015) following the octanoyl-l-carnitine, malate, ADP, glutamate, and pyruvate as substrates for CI (FAO, Fig 1B). Tissue exposed to the Dauno-HF plasma showed 33% lower maximal OXPHOS (P = 0.0035) and 30% lower maximal uncoupling (P = 0.002) than the tissue exposed to the Dauno-Control blood plasma following the glutamate, malate, ADP, and pyruvate as substrates for CI (Fig 1C). The maximal OXPHOS and the maximal uncoupling of tissue exposed to the Dauno-HF blood plasma were also 29% (P<0.0001) and 22% (P = 0.0001) lower than the tissue exposed to the Dauno-Control blood plasma following the octanoyl-l-carnitine, malate, ADP, glutamate, and pyruvate as substrates for CI (FAO, Fig 1D). The overall result indicates that HF blood plasma significantly decreased cardiac tissue respiration irrespective of HF etiology.

### Effects of HF blood plasma on the respirometry of cardiac cell lines

**Fig 2:**
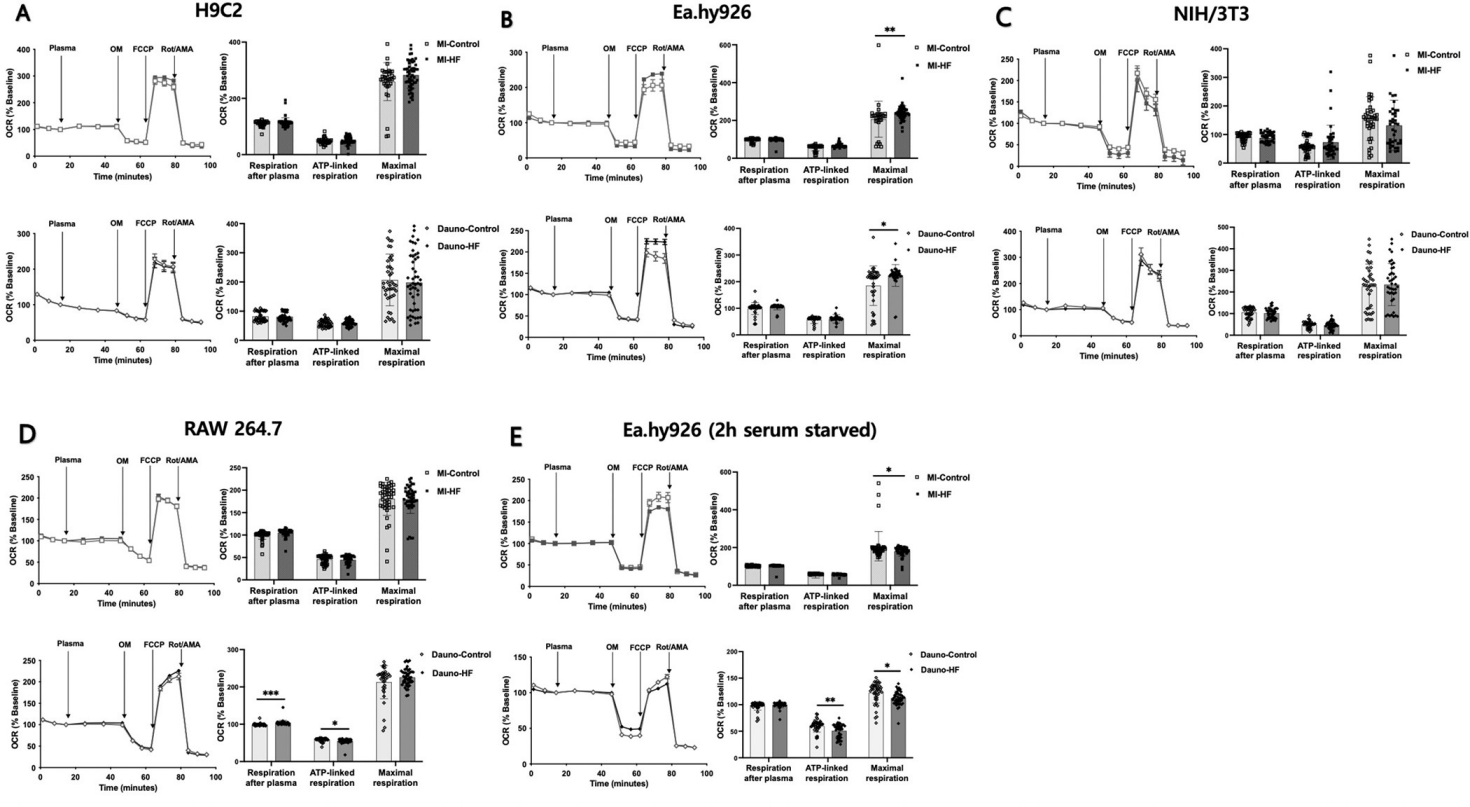
Mitochondrial stress test to assess oxygen consumption rates in cardiac cell types exposed to heart failure rabbit blood plasma. Baseline-corrected oxygen consumption rate (OCR) traces of mitochondrial stress tests in the Seahorse extracellular flux analysis system on H9C2 myoblast (A), Ea.hy926 endothelial cells (B), NIH/3T3 fibroblasts (C), and RAW 264.7 macrophage (D) without serum starvation, and Ea.hy926 endothelial cells, 2 h serum-starved before the assay (E). After 3 measures of baseline OCR, the cells were exposed to the blood plasma from the heart failure rabbits or corresponding control rabbits for 30 min with 3 measures of OCR. Then, sequential addition of oligomycin, FCCP, and rotenone, and antimycin A was followed to determine ATP-linked respiration, maximal respiration, and non-mitochondrial respiration, respectively (left panels of each image, normalized to the baseline values). Quantified baseline-corrected OCR upon the plasma exposure, ATP-linked respiration, and maximal respiration at each cell type was compared between the HF and corresponding control group (right panels of each image). Assays utilized n = 6–8 plasma samples per group and 6–7 technical replicates per each plasma sample. Data are presented as means and SEM. Two-way ANOVA with Bonferroni post-hoc test was used. *P < 0.05, **P < 0.01, ***P < 0.001, ****P < 0.0001 of HF vs. Control. ATP = adenosine triphosphate; Dauno = Daunorubicin; FCCP = carbonyl cyanide *p* trifluoro methoxyphenylhydrazone; HF = heart failure; MI = myocardial infarction; OM = oligomycin; Rot /AMA = rotenone/antimycin A.

Given the decreased mitochondrial function of mouse LV tissue upon acute exposure of HF rabbit blood plasma, we subsequently assessed the impact of blood plasma on the mitochondrial respiration in cardiac component cell lines using the *Seahorse* extracellular flux analyzer. The mito-stress test revealed that the addition of both types of HF blood plasma did not alter the mitochondrial function of H9C2 myoblasts (Fig 2A) or NIH/3T3 fibroblasts (Fig 2C), compared to the corresponding control blood plasma. The RAW 264.7 macrophage cells exposed to Dauno-HF blood plasma showed 5.02% higher OCR after plasma exposure (P = 0.0004) and 5.30% lower ATP-linked respiration (P = 0.0392), compared to the cells exposed to Dauno-Control blood plasma, while the effects of MI-HF and MI-Control blood plasma were comparable (Fig 2D). In the Ea.hy926 endothelial cells, both MI-HF and Dauno-HF plasma resulted in 15.84% and 19.16% higher maximal respiration (P = 0.0012 and 0.0112, respectively, Fig 2B). Given the consistent changes in the mitochondrial function of Ea.hy926 endothelial cells by plasma from both HF types, we further performed mito-stress tests with the same protocol on the Ea.hy926 after serum starvation for 2 h (Fig 2E). As a result, in contrast to the first assay without serum starvation, the MI-HF plasma resulted in 13% lower maximal respiration (P = 0.0259), and the Dauno-HF plasma resulted in 16% lower ATP-linked respiration (P = 0.0022) and 8% lower maximal respiration (P = 0.02) of Ea.hy926 endothelial cells, compared to the corresponding control blood plasma.

### Effects of HF blood plasma on endothelial cell permeability

**Fig 3:**
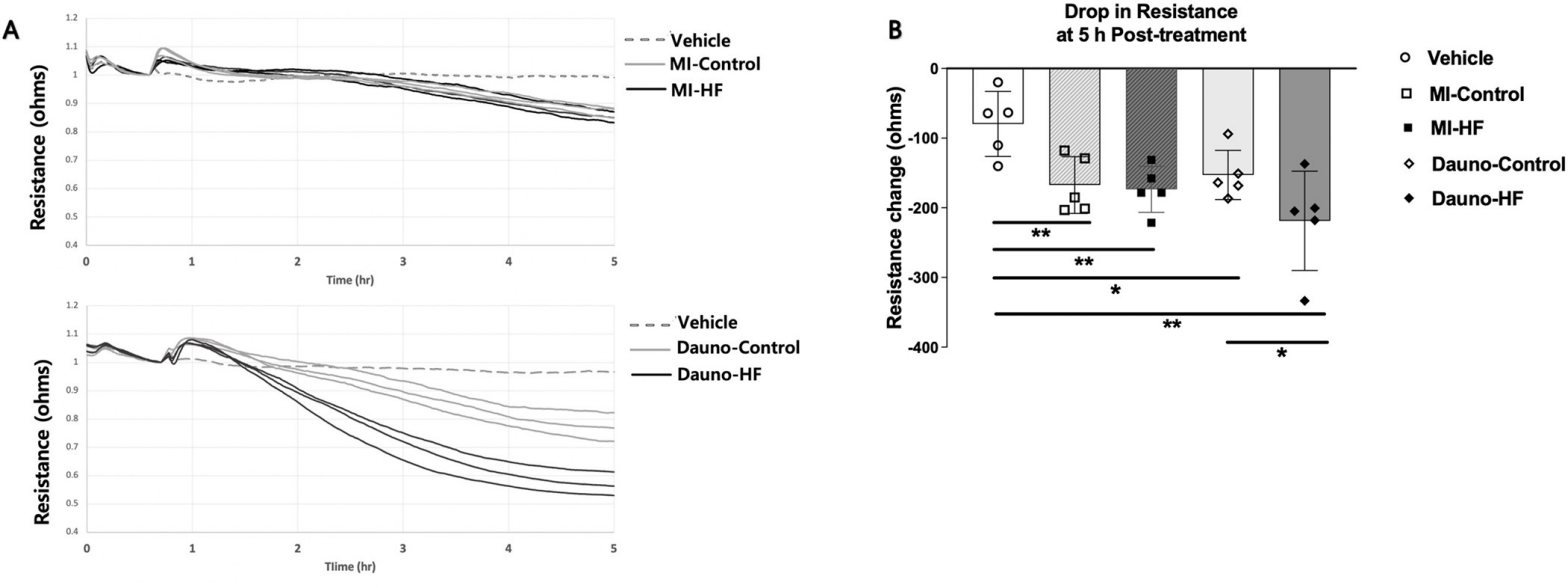
Endothelial cell permeability after exposure to daunorubicin heart failure rabbit blood plasma. Rat heart microvascular endothelial cells were seeded to confluence, and monolayer permeability was measured by electric cell-substrate impedance sensing (ECIS). (A) Representative tracings of cell monolayers exposed to treatments. Cells were equilibrated and then exposed to treatment (arrow indicates at the point the treatment was added), and resistance was measured for ~24h. Representative tracings show the monitoring of the cells for 10h and are presented as the resistance normalized to the point before the addition of treatment (arrow). Both panels were treated with vehicle (1% PBS) or 1U/ml thrombin as negative and positive controls, respectively. Upper panel treatments include MI-control and MI-HF, and bottom panel treatments include Dauno-control and Dauno-HF. (B) Quantification of averaged changes in monolayer resistance at 5 h after treatment in each group. Assays utilized n = 5 plasma samples per group and 3 technical replicates per each plasma sample. Data are presented as means and STD. Unpaired Student’s t-test was used. *P < 0.05, **P < 0.01 of vehicle vs. blood plasma and HF vs. Control. Dauno = Daunorubicin; HF = heart failure; MI = myocardial infarction.

We then evaluated the influence of HF blood plasma on the barrier function of endothelial cells (Fig 3A). The blood plasma from both HF types produced a significantly greater decrease in monolayer resistance of the rat heart microvascular endothelial cells at 5 h-treatment, compared to the vehicle (PBS) treatment, (P = 0.0028, P = 0.0013, P = 0.0043, P = 0.0120 for the vehicle vs. MI-HF, MI-Control, Dauno-HF, and Dauno-Control, respectively), which indicates that permeability of endothelial cells exposed to the HF blood plasma was increased (Fig 3B). The cell monolayer treated with MI-HF blood plasma and the MI-Control blood plasma showed similar decreases in resistance, and MI-HF-induced endothelial barrier function was not significantly different from MI-control. On the other hand, the resistance of cell monolayers treated with Dauno-HF blood plasma was significantly decreased at 5 h-treatment, compared to the cells treated with Dauno-Control blood plasma (P = 0.0345), demonstrating that endothelial permeability was increased by exposure to the Dauno-HF blood plasma.

### Effects of HF blood plasma on endothelial cell viability

**Fig 4:**
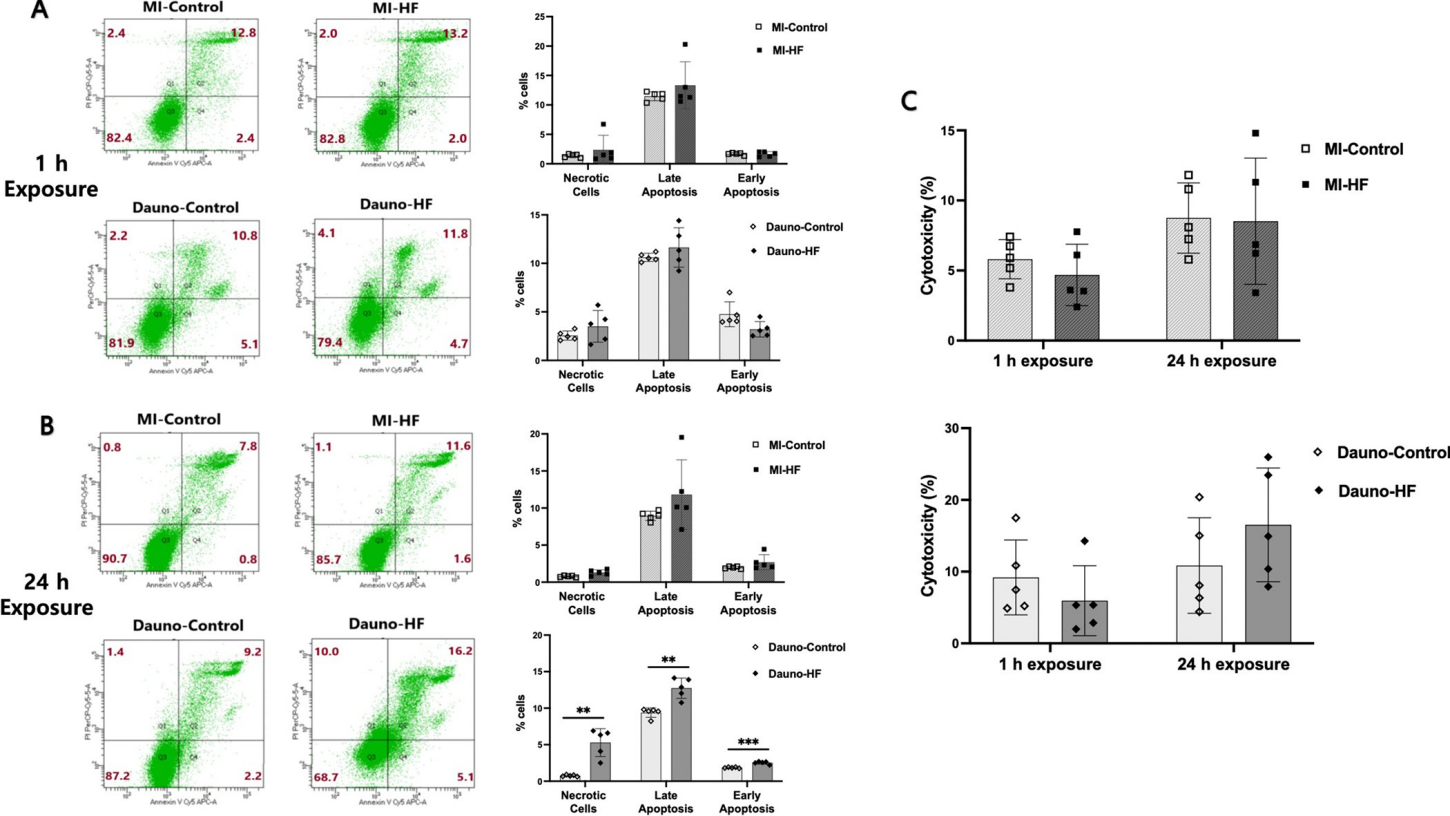
Viability of endothelial cells exposed to heart failure rabbit blood plasma. Representative scatter plot flow cytometry of Ea.hy926 endothelial cells exposed to rabbit blood plasma for 1 h and 24 h, followed by staining with annexin-V and propidium iodide (PI) to detect apoptosis or necrosis at 1 (A) and 24 h (B) post exposure and comparison of the quantified proportion of early apoptotic cells (Annexin V+/PI-), late apoptotic cells (Annexin V+/PI+), and necrotic cells (Annexin V-/PI+) between those exposed to HF and corresponding control blood plasma. Assays utilized n = 5 plasma samples per group and 3 technical replicates per each plasma sample. Data are presented as means and SEM. Unpaired Student’s t-test was used. *P<0.05 and **P<0.01 in comparing the HF versus Control blood plasma. (C) LDH assay cytotoxicity detected in Ea.hy926 cells exposed to the HF and the corresponding control blood plasma for 1 h and 24 h, respectively. A % cytotoxicity was defined as (Plasma-treated LDH activity—Spontaneous LDH activity)/(Maximum LDH activity-Spontaneous LDH activity) x 100 (%). Assays utilized n = 5 plasma samples per group and 3 technical replicates per each plasma sample. Data are presented as means and SEM. Dauno = Daunorubicin; HF = heart failure; LDH = lactate dehydrogenase; MI = myocardial infarction.

Annexin V/PI double staining was used to assess effects of rabbit blood plasma on the viability of Ea.hy926 cells. Quantification of the flow cytometry results demonstrated that exposure to the MI-HF and Dauno-HF blood plasma for 1 h did not induce greater cell death than the corresponding control blood plasma (Fig 4A). Exposure to the MI-HF blood plasma for 24 h also influenced the endothelial cell viability similarly to the exposure to the MI-Control blood plasma (Fig 4B). On the contrary, cells exposed to the Dauno-HF blood plasma for 24 h showed significant increases in annexin-V-/PI+ stained (i.e., necrotic) cells and annexin-V+/PI+ stained (i.e., late apoptotic) cells than those exposed to the Dauno-Control blood plasma (Fig 4B. mean % of necrotic cells; 5% vs. 0.75%, P = 0.0095; mean % of late apoptotic cells; 13% vs. 9%, P = 0.023)

In the LDH release assay to test the cytotoxicity of HF blood plasma on the Ea.hy926 endothelial cells, we did not observe any significant differences in the % of cytotoxicity between the effects of the HF and the Control blood plasma in both of 1 h and 24 h exposure. However, there was a trend toward a higher cytotoxic effect after exposure to the Dauno-HF blood plasma for 24 h, compared to the Dauno-Control blood plasma (16.52% vs. 10.86%, P = 0.0603, Fig 4C).

### Effects of HF blood plasma on endothelial cell mitochondrial proteins and morphology

**Fig 5:**
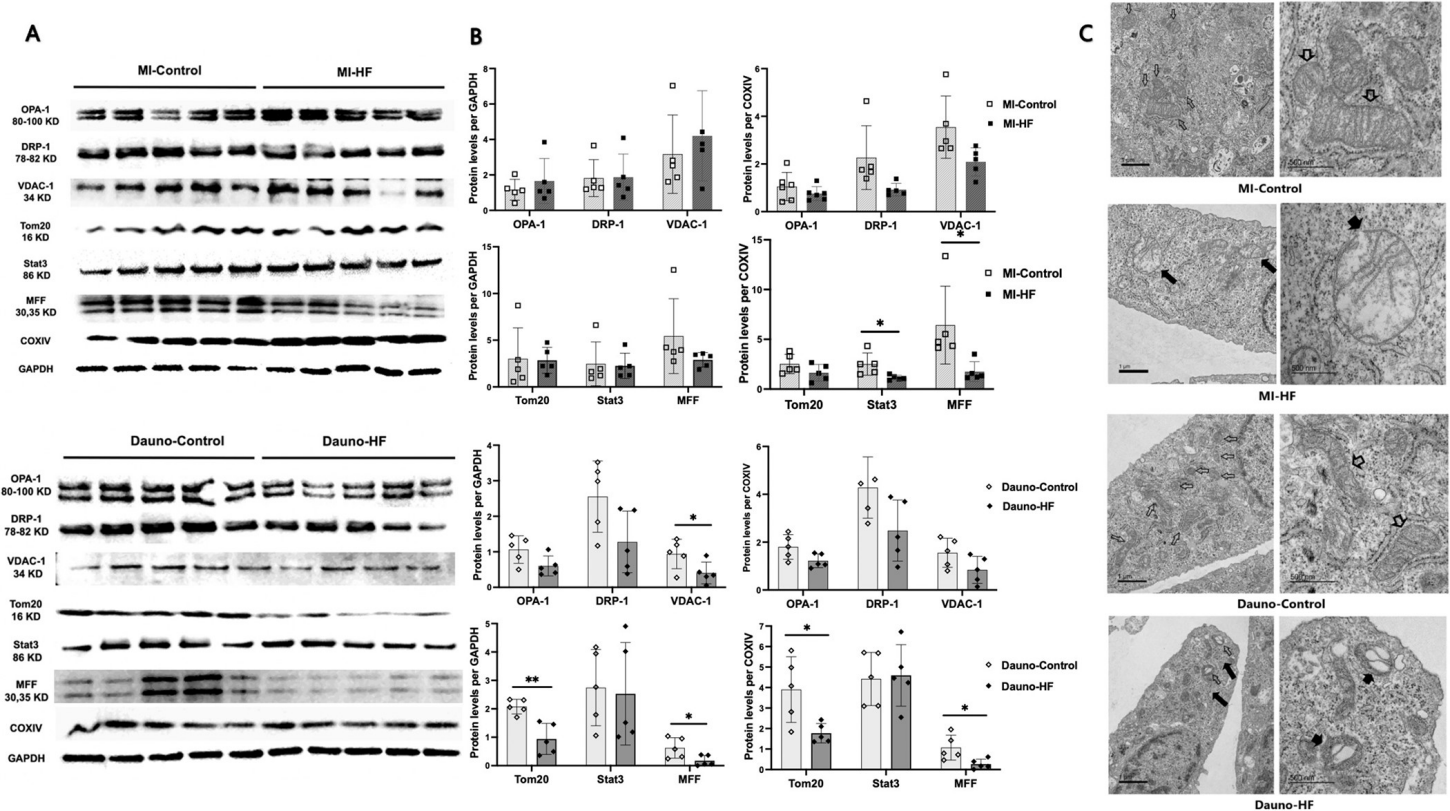
Mitochondrial protein and ultrastructural changes in endothelial cells exposed to heart failure rabbit blood plasma. Ea.hy926 endothelial cells were grown and serum-starved for 2 h, followed by exposure to the rabbit blood plasma for 1 h. Proteins were purified and subjected to western blot analysis. Representative blots (A) of samples exposed to five blood plasma from each group: MI-HF versus MI-control and Dauno-HF versus Dauno-control. GAPDH and COX IV were used as total protein loading control and mitochondrial loading control, respectively. Band intensity quantification (B) of the western blot normalized to GAPDH and COXIV. Assays utilized n = 5 plasma samples per group and 3 technical replicates per each plasma sample. Data are presented as means and SEM. Unpaired Student’s t-test was used. *P<0.05 and **P<0.01 in comparing the HF versus Control blood plasma. (C) Mitochondrial ultrastructural changes in endothelial cells exposed to HF rabbit blood plasma were determined. Ea.hy926 endothelial cells were grown and serum-starved for 2 h, followed by exposure to the rabbit blood plasma for 1 h. Narrow arrows indicate characteristic mitochondria in the representative cells from each group. Compared to the cells exposed to the MI-Control blood plasma showing normal mitochondrial morphology, those exposed to the MI-HF blood plasma show swollen, balloon-like mitochondria cristae disarrangement, partial cristolysis, and electron-lucent matrix. Cells exposed to the Dauno-Control blood plasma shows normal mitochondrial morphology, while the mitochondria of cells exposed to the Dauno-HF blood plasma shows swelling with loss of cristae and effacement of central architecture with the balloon-like, vesicular structures. Thick black arrows indicate lysed or ruptured outer membrane of mitochondria exposed to the HF blood plasma. Scale bars: 1 μm (left-side images) and 500 nm (right-sided images). Assays utilized n = 5 plasma samples per group and 3 technical replicates per each plasma sample. COXIV = Cytochrome C oxidase; Dauno = Daunorubicin; HF = heart failure. MI = myocardial infarction.

Western blot analysis showed significant impact of HF blood on protein expression of several important components of mitochondrial membranes (Fig 5A). When the band intensity was normalized to GAPDH, expression levels of VDAC-1, Tom20, and MFF were significantly lower in the cells exposed to Dauno-HF blood plasma compared with those exposed to Dauno-Control blood plasma (Fig 5B). After COX IV normalization, the expression levels of Tom20 and MFF were significantly lower in the cells exposed to Dauno-HF blood plasma compared with those exposed to Dauno-Contol blood plasma, while the level of Stat3 was significantly lower in the cells exposed to MI-HF blood compared with those exposed to MI-Control blood. Because we found a consistent impact of MI-HF and Dauno-HF blood plasma on mitochondrial function in Ea.hy926 endothelial cells only, we proceeded with further analysis of this cell type. Ultrastructural analysis of mitochondria in Ea.hy926 cells exposed to blood plasma was performed using transmission electron microscopy. The left and the right panels of Fig 5C display representative images of the cells from each group at 8000x and 25000x magnification, respectively. In the cells exposed to the MI-Control blood plasma and Dauno-Control plasma, the mitochondria exhibited well-defined double membranes with normal or minimal loss of cristae arrangement and matrix material. However, the mitochondria of cells exposed to the MI-HF plasma had severe morphological deformations, including swelling with the loss of cristae and matrix, and empty spaces. A rupture of the outer mitochondrial membrane was also observed. The cells exposed to the Dauno-HF plasma also showed swollen mitochondria with the loss of cristae and matrix, effacement of central architecture with the balloon-like, vesicular structures, and widespread disruption of the outer membrane.

## Discussion

Our findings identify endothelial cells and mitochondria as targets to be probed in response to HF blood markers to help define disease mechanisms and develop therapeutics. A role for cardiac endothelium in the pathophysiology of HF has been increasingly recognized. Beneficial endothelium-regulated process in the heart including protective barrier function, nutrient delivery, and release of paracrine factors to maintain cardiac contractility are disrupted upon endothelial cell damage induced by HF [10, 11]. Endothelial cells provide a barrier between the blood and cardiac tissue. Endothelial damage occurs during HF progression and induces inflammation, oxidative stress, and altered nitric oxide bioavailability, thereby aggravating disease severity [12, 13]. Previous studies demonstrated that congestive HF and blood from patients with acute myocardial infarction (MI) induced apoptosis of endothelial cells associated with the mitochondrial cytochrome c release [14, 15]. Moreover, pro-inflammatory cytokines and reactive oxygen species, are produced by endothelial cells in the failing heart and target cardiomyocytes, thereby worsening the HF [10, 16]. However, evidence for the mechanistic interaction between factors in plasma from HF patients and blood endothelial cell perturbation is scarce. The present study, to our knowledge, is the first to demonstrate effects of blood from distinct HF animal models on the endothelial cells in terms of barrier function, metabolism, and death.

Relative impermeability of cardiac microvascular endothelium associated with the presence of tight junctions prevents the myocardium from exposure to toxins [17, 18]. Thus, it may be natural that compromised endothelial integrity in HF should result in compromised cardiomyocyte function with harmful compounds circulated through blood vessels that aggravate disease. Increased permeability of endothelial cells by incubation with Dauno-HF blood in the current study may provide consistent evidence on the initiating action of endothelium on the development of HF. A previous ex vivo study revealed that human umbilical vein endothelial cells treated with blood serum from the sepsis patients showed increased endothelial monolayer permeability in relation to the elevated serum level of high mobility group box (HMGB)-1 [19] that is known to be increased in the bloodstream of HF patients as well [20]. A recent clinical study demonstrated that blood levels of angiopoietin-2, an indicator of vascular permeability, was increased in patients with non-ischemic HF, while the levels were comparable between the healthy volunteers and patients with ischemic HF [21]. The latter report provides mechanistic insight and may also explain the discordance of results in the current study that showed maintenance of integrity of endothelial cells exposed to MI-HF blood. Conflicting results between the effects of blood from the MI and daunorubicin induced HF were also seen in the apoptosis assays in our studies. The cells incubated with Dauno-HF blood showed significantly increased late apoptosis and necrosis, which confirms the previous report that showed increased apoptosis of human umbilical vein endothelial cells by incubation with the blood serum of HF patients [14, 22]. On the contrary, cells incubated with MI-HF blood did not show increased apoptosis or necrosis. We could assume that distinct pathophysiological processes according to the etiology of HF could result in different types and levels of blood circulating factors that participate in endothelial cell injury. Blood from the daunorubicin-induced HF model may retain more harmful factors that transfer to cardiac endothelium compared with blood from ischemic HF, although there has been no direct evidence to support this, which calls for further studies to specify relevant blood factors according to the type of HF.

Instead of the well-known blood factors, such as HMGB-1, pro-inflammatory cytokines, and reactive oxygen and nitrogen species, we focused on the mitochondria as a potential mediator of endothelial deterioration in response to HF plasma. A role of endothelial mitochondria has been largely neglected in the past because large proportions of ATP in endothelial cells are normally generated through anaerobic glycolysis [23]. On the other hand, many other aspects of mitochondrial biology in endothelial cells have recently been emerging. Endothelial mitochondria integrate environmental cues and modify cell biogenesis, dynamics, and programmed degradation, thereby regulating the cellular homeostasis and function [24, 25]. Mitochondria are reported to play a central role especially in the cell signaling for the regulation of cell death [24]. Alterations in cellular expression level of some mitochondrial proteins induced by HF blood in the current study may provide mechanistic insights as well. MFF, one of the mitochondrial outer membrane proteins, was significantly down-regulated on the cells exposed to Dauno-HF blood. It is a receptor-like protein for recruitment of cytoplasmic dynamin-related protein (Drp)-1 to the mitochondria to mediate fission [26]. A large body of evidence highlights that appropriate mitochondrial fission is a key cytoprotective mechanism to promote a clearance of damaged components in response to various stress states [24]. A recent study using a sepsis model demonstrated that overexpression of Drp-1 not only upregulated mitophagy but also prevented endothelial hyperpermeability and apoptosis [27]. Although the Drp-1 was not significantly affected by HF blood in the current study, a very low expression of MFF could have interrupted proper fission processes, thereby leading to loss of barrier integrity and death of endothelial cells. Reduced expression level of Tom20 could also have contributed to deterioration of the endothelial cells after exposure to Dauno-HF blood. Tom20, a mitochondrial transmembrane protein, is an element of protein import machinery which controls recognition, translocation, and sorting of mitochondrial components [28]. Tom20 also interacts with respiratory chain complexes and membrane architecture, and thus, is indispensable for maintenance of bioenergetics, membrane dynamics, and quality control of the mitochondria [29]. Moreover, dysfunctional mitochondrial import causes accumulation of precursor proteins in the cytosol, thereby leading to cell death. A recent experimental study of Parkinson’s disease demonstrated a significant relationship between lower expression of Tom20 and impaired oxidative phosphorylation and accumulation of aggregated proteins inside mitochondria [30], which is consistent with our finding. Another protein that was down-regulated in the cells exposed to Dauno-HF blood is the VDAC-1, a mitochondrial outer membrane channel. VDAC-1 regulates bioenergetic function and interaction between the mitochondria and the rest of the cell [31]. Moreover, VDAC-1 can serve to regulate life and death decisions of the cells, although evidence is confusing and contradictory [32]. The western blot results in the current study which showed significant down-regulation of several essential mitochondrial membrane proteins by exposure to Dauno-HF blood may point the mitochondria as a likely catalyst that promotes endothelial cell death and consequent HF progression.

Bioenergetic function of endothelial mitochondria, which was underestimated in the past, has recently emerged as an important modulator of cell homeostasis. A previous study on bovine aortic endothelial cells revealed the presence of meaningful reserve respiratory capacity of mitochondria that is available when demand is increased in response to oxidative and nitrosative stress [33]. In our first mito-stress test on the Ea.hy926, exposure to the HF plasma may have impaired glycolysis, which subsequently could have led to increased oxidative phosphorylation of mitochondria to compensate for the energy shortage. Mito-stress on the cells starved for 2 more hours resulted in significantly lower maximal respiration of cells exposed to the HF blood, which was contrary to the former result. Starvation could have made the cells rely more on mitochondrial energy production [34], which might be impaired by pathogenic contents present in the HF blood thus unmasking certain aspects of endothelial mitochondrial responses to stress. It is likely that down-regulation of VDAC-1, MFF, and Tom20 in the cells exposed to the Dauno-HF blood might have attributed to the impaired bioenergetic function of mitochondria as well. On the other hand, none of these proteins were significantly down-regulated on the cells exposed to the MI-HF blood, while protein expression of Stat3 normalized by COXIV to control the mitochondrial fraction only was significantly reduced in this cell group. The Stat3, an important regulator of crosstalk between the cell populations in the heart, has a multitude of distinct function to promote occurrence of either pathological or protective process in the cardiomyocyte and endothelium [35, 36]. The mitochondrial Stat3 was revealed to serve as a key modulator of cellular respiration a decade ago. In Stat-/- cells and knock-out mice, activities of complexes I and II of the mitochondrial electron transport chain were significantly decreased, indicating that Stat3 is required for optimal respiration [37]. MI-HF blood could have affected it, although this impact on the bioenergetics was not linked to the barrier function and cell death.

Our study results may provide clinical implication of endothelial cells in diagnosis and targeted therapy of HF by applying such cells and biology as screening approaches. In addition, we saw the possibility that cardiac tissue metabolism assessed by O2K may be able to discern the HF etiology, since the analysis revealed an increase of FAO in the tissue exposed to MI-HF blood while the tissue exposed to Dauno-HF blood showed a decreased FAO. Several fatty acids and sugar alcohol contents which were elevated in the blood plasma of MI-HF rabbits [8] could have promoted enhanced FAO of myocardial tissue [38, 39]. Meanwhile, strategies to maintain endothelial integrity may offer a novel therapeutic opportunity in HF management. Indeed, treatment of simvastatin on the human cardiac microvascular endothelial cells attenuated daunorubicin-induced hyperpermeability through increased tight junction formation [12]. So far, therapeutic approaches in HF directed at endothelial cell dysfunction have focused only on the restoration of vasodilation [40]. Our finding may suggest the need to consider endothelial cell barrier function as a target for comprehensive HF management.

There are some limitations to be noted in this study. First, the blood plasma, samples of cardiac tissue, and various cell lines were derived from different species. Our studies are limited as there is potential for inter-species interactions. Our initial aim was to assess the acute effects of plasma factors from HF models in non-intact systems. However, we cannot fully exclude the potential influence of certain biological reactions on the results of the experiments. We observed that the blood of the control rabbit group reduced endothelial integrity greater than the vehicle, which could be partly attributable to interactions between the different species. Also, there is potential for immune reactions to be elicited by the blood plasma itself, but as our systems for assays utilize cell or tissue these immune reactions are less likely to be present to confound the experiments. Using control plasma also allowed us to assess relative changes to account for the baseline effect of plasma on our assays. Second, we used four immortalized cell lines that are not derived from cardiac tissue for the Seahorse flux analysis due to the practical difficulty in obtaining all the cell types from the heart. Since cellular metabolism varies somewhat depending on the type of organ the cells originate from, our data may not completely reflect the actual responses of cardiac cellular populations. Third, the endothelial cell line used in most of the experiments of our study, Ea.hy926, is derived from umbilical vein, not from heart microvasculature. Thus, some discrepancies between the current result and the potential pathological impact of blood plasma on the cardiac vasculature cannot be ruled out. Differences in endocardial and capillary endothelium in terms of cell shape, cytoskeletal organization, and electrochemical properties [41] may lead to a different response to HF blood, which needs to be further elucidated in future studies.

## Conclusions

We demonstrate in the current study that respiration of myocardial tissue and endothelial cells are strongly affected by blood plasma derived from daunorubicin-induced and MI-induced HF rabbits. The blood of daunorubicin-induced HF rabbits also disrupted endothelial monolayer integrity and increased late apoptosis and necrosis of endothelial cells. Our findings suggest a novel mechanistic paradigm that endothelial cells may be a primary target whereby HF pathology manifests and accelerates. Alterations in mitochondrial membrane proteins provide evidence for a potential role of mitochondria in the deterioration of endothelial metabolic and barrier function and cell death in HF. Therefore, targeting endothelial cell mitochondria that have been relatively under-studied in HF, may aid in HF therapy. Future work to define the circulating factors in HF patients, such as HMGB1, pro-inflammatory cytokines, reactive oxygen nitrogen stress, and metabolites, in relation to endothelial function and survival are warranted to support the current result.
